# Etiology and Prognosis of Cardiogenic Shock in a Secondary Center without Surgical Back-Up

**DOI:** 10.1155/2019/3869603

**Published:** 2019-12-09

**Authors:** Laurent Bonello, Marc Laine, Etienne Puymirat, Victoria Ceccaldi, Mélanie Gaubert, Franck Paganelli, Pr Franck Thuny, Thibaut Dabry, Guillaume Schurtz, Clement Delmas, Julien Mancini, Gilles Lemesle

**Affiliations:** ^1^Assistance Publique-Hôpitaux de Marseille, Intensice Care Unit, Hôpital Nord, Marseille, France; ^2^Mediterranean Academic Association for Research and Studies in Cardiology (MARS Cardio), Marseille, France; ^3^Aix-Marseille University, INSERM UMRS 1076, Marseille, France; ^4^Département de Cardiologie, Hôpital Européen Georges Pompidou, Assistance Publique des Hôpitaux de Paris, Paris, France; ^5^USIC et Centre Hémodynamique, Institut Cœur Poumon, Centre Hospitalier Régional et Universitaire de Lille, Faculté de Médecine de l'Université de Lille, INSERM UMR1011, Lille F-59000, France; ^6^Intensive Cardiac Care Unit, Rangueil University Hospital, Toulouse, France; ^7^Institute of Metabolic and Cardiovascular Diseases (I2MC), Institut National de la Santé et de la Recherche Médicale (INSERM), UMR-1048, Toulouse, France; ^8^Aix-Marseille Univ, INSERM, IRD, UMR912, SESSTIM, “Cancers Biomedicine & Society” Group, Marseille, France; ^9^APHM, Timone Hospital, Public Health Department (BIOSTIC), Marseille, France

## Abstract

**Background:**

Cardiogenic shock (CS) remains a major challenge in contemporary cardiology. Data regarding CS etiologies and their prognosis are limited and mainly derived from tertiary referral centers.

**Aims:**

To investigate the current etiologies of cardiogenic shock and their associated short- and long-term outcomes in a secondary center without surgical back-up.

**Methods:**

We performed an observational prospective monocenter study. All patients admitted for a first episode of CS related to left ventricular dysfunction were enrolled. The definition of CS was consistent with the European Society of Cardiology guidelines. Patients were followed for 6 months. Etiologies were analyzed, and survival rates derived from Kaplan-Meier estimates were compared with the log-rank test.

**Results:**

Between January 2015 and January 2016, 152 patients were included. The first most common cause of CS was acute decompensation of chronic heart failure (CHF). Acute coronary syndromes (ACS) were the second most common cause of CS (35.4%). At one month, the all-cause mortality rate was 39.5% and was similar between ACS and CHF (43% vs 35%, respectively; *p*=0.7). In a landmark analysis between 1 and 6 months, we observed a significantly higher mortality in patients with CHF than in patients with ACS (18% vs. 0%; *p*=0.01).

**Conclusions:**

In the present registry, acute decompensation of chronic heart failure was the most common cause of CS, while ACS complicated by CS was the second most common cause. Of importance, acute decompensation of CHF was associated with a significantly worse outcome than ACS in the long term.

## 1. Introduction

Cardiogenic shock (CS) remains one of the greatest challenges in cardiology. CS is caused by various conditions that can affect the right and/or the left ventricle (LV); however, the most common causes are related to LV dysfunction. The prevalence of CS is stable, while the prognosis remains striking, with a 40 to 50% death rate at 30 days [1, 2]. Studies involving patients with CS are scarce, and most data are provided by registries. The CARDSHOCK registry was a multicenter prospective study assessing the etiology and prognosis of CS. In this registry, acute coronary syndromes (ACS) were responsible for 81% of CS [3]. However, registries yielded inconsistent reports on the etiologies of CS [1, 3]. In addition, the FAST MI registry observed a reduction in CS following ACS, which was associated with the wide use of early reperfusion [4]. However, recent advances in chronic heart failure related to LV dysfunction have improved the prognosis of these patients [5]. Finally, most studies on CS are performed by tertiary centers with surgical back-up, possibly causing a selective skew related to the varying referral patterns of patients to these centers, which depend on the etiology of CS and the patients' baseline characteristics, such as age or comorbidities [3]. Thus, identifying the characteristics of unselected patients with CS admitted to a secondary center that lacks surgical back-up or a transplant program would be of interest. In addition, since shock centers are supported by the recent literature, determining the outcome of CS patients in secondary centers may help determine if they should be allowed to become shock centers [6]. We therefore aimed to prospectively investigate the etiologies of CS and the associated long-term prognosis in the contemporary era in our secondary center.

## 2. Methods

We performed a single-center prospective observational study including all patients admitted for a first episode of CS related to LV dysfunction between January 2015 and January 2016 in our institution.

The hospital Nord of Marseille (France) is an academic institution with a large emergency department but without on-site cardiac surgery or transplant programs. It is part of a regional network of care centered on a tertiary center with surgical back-up. Informed consent was obtained from each patient before inclusion in the study, and the study protocol conformed to the ethical guidelines of the 1975 Declaration of Helsinki as reflected in a priori approval by the institution's Human Research Committee. CS and its etiologies were assessed by 2 independent cardiologists (LB and ML) based on clinical, biological, electrocardiographic, echocardiographic, and imaging data according to definitions listed in guidelines [7]. A third cardiologist was consulted in cases of disagreement (MP).

The inclusion criteria required a systolic blood pressure of <90 mmHg (after adequate fluid infusion) for 30 min or the need for vasopressor therapy to maintain a systolic blood pressure >90 mmHg and signs of peripheral hypoperfusion (altered mental status/confusion, cold periphery, oliguria <0.5 mL/kg/h, or blood lactate >2 mmol/L) according to the usual definition of CS [7].

The exclusion criteria included refusal to participate, CS that occurs following a cardiac procedure, and a history of CS.

### 2.1. Therapeutic Strategy

For all patients, a similar protocol based on ESC guidelines was used; dobutamine infusion with norepinephrine was used in cases of persistent hypotension. Fluid management was performed based on clinical findings and noninvasive monitoring with transthoracic echocardiography. In addition, etiologic treatment was provided when required, including primary PCI in ACS or cardioversion in cases of refractory arrhythmias.

The primary outcome was the rate of all-cause death at 1 month. The secondary endpoints were the rate of death at 6 months (landmark analysis was conducted because the etiologies investigated were expected to have different effects across those time periods) and the rate of CS recurrences at 6 months.

Outcomes were collected during follow-up with direct patient contact either during out-patient clinic visits or telephone interviews. In cases where the patient could not be reached, the general practitioner and family members were reached to assess vital status.

### 2.2. Statistical Analysis

The results are presented as numbers (*n*) and percentages (%), means and standard deviations (SD), or median and interquartile range (IQR) for variables with a skewed distribution. Between-group comparisons were performed using Student's *t*-test or Mann–Whitney *U* test, as appropriate.

CS recurrence rates were compared with Fischer's exact tests. Survival rates were derived as Kaplan–Meier estimates and compared with the log-rank test. To compare the secondary endpoints, a landmark analysis was conducted because the etiologies investigated were expected to have different effects across those time periods. A *p* value <0.05 was considered significant.

## 3. Results

### 3.1. Baseline Characteristics

Over 1 year, 152 patients with a first occurrence of CS were admitted to our intensive care unit. The main characteristics of the cohort are described in Table 1. The mean age was 71.9 ± 12.7 years, and half of the patients had a history of previous acute heart failure. The mean LV ejection fraction on admission was low (26.5 ± 10.5%).

On admission, the mean arterial pressure was low at 62 ± 11 mmHg, and all patients exhibited signs of peripheral organ hypoperfusion. Regarding biologic data, lactate levels were high: 4.3 ± 3.5 mmol/l.

### 3.2. Etiologies of Cardiogenic Shock

**Figure 1:**
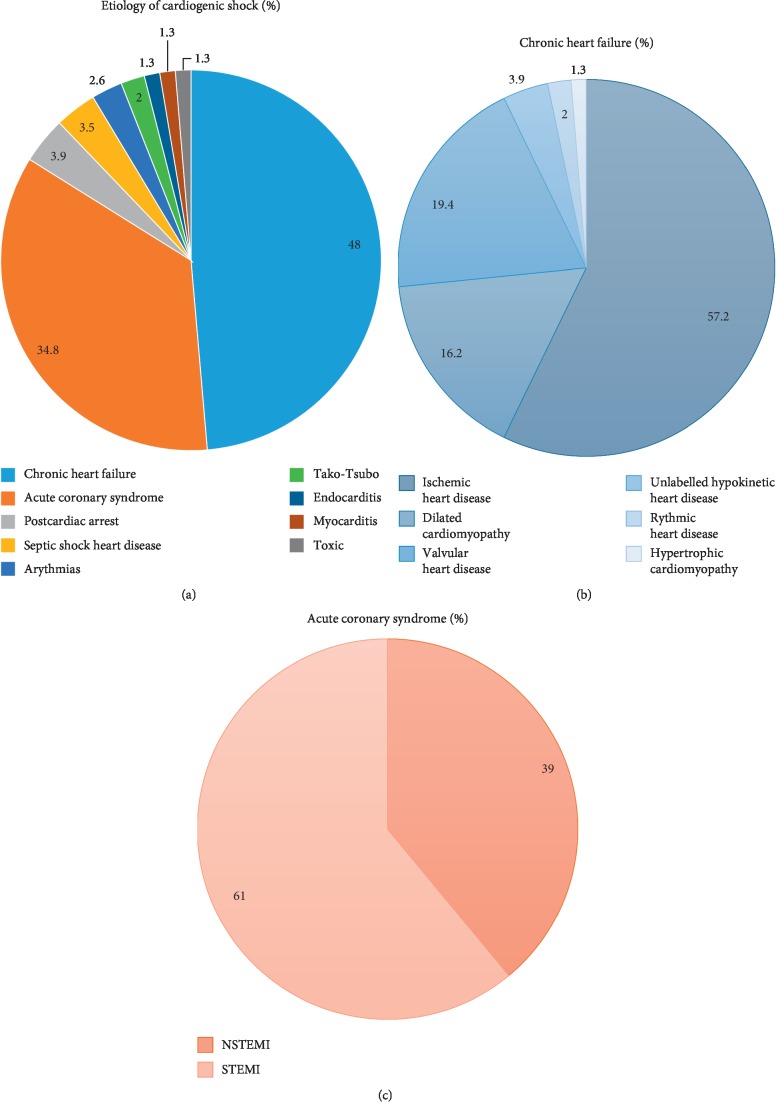
Panel (A), etiologies of CS; Panel (B), underlying cause of chronic heart failure; Panel (C), type of acute coronary syndrome.

The etiology of CS was identified in 98.7% of patients during their hospital stay. The etiologies are detailed in Figure 1. The main etiology of CS was acute decompensation of chronic heart failure (CHF) with reduced LV function in 46.7% of cases. ACS was the second etiology (34.8%), including ST elevation (61%) or non-ST elevation (39%) ACS. Other causes accounted for less than 20% of CS cases.

### 3.3. Drugs

Management of CS patients is described in Table 2. Almost all patients received dobutamine (90.8%). Norepinephrine was required in addition to dobutamine in more than half of the patients (52.6%). Epinephrine was less frequently used (27%). Diuretics were administered in 69.7% of cases, and dialysis was required in 11.2% of patients. The mean duration of catecholamine support was 7.8 ± 6.8 days. Mechanical circulatory support with ECLS was required in 5.9% of patients, and an intra-aortic balloon pump was required in 3.3% of patients. Invasive ventilation was used in 34.1% of patients because of refractory hypoxemia.

### 3.4. In-Hospital Complications

The rate of in-hospital complications was high. Almost half of the patients suffered either localized or generalized sepsis during their hospital stay (43.4%). Acute kidney failure and transfusions were also frequent (32.2 and 8.6%, respectively) (Table 3). The mean duration of in-hospital stay was 18 ± 15.4 days, including 9.33 ± 7.51 days in the ICU.

### 3.5. Outcomes

**Figure 2:**
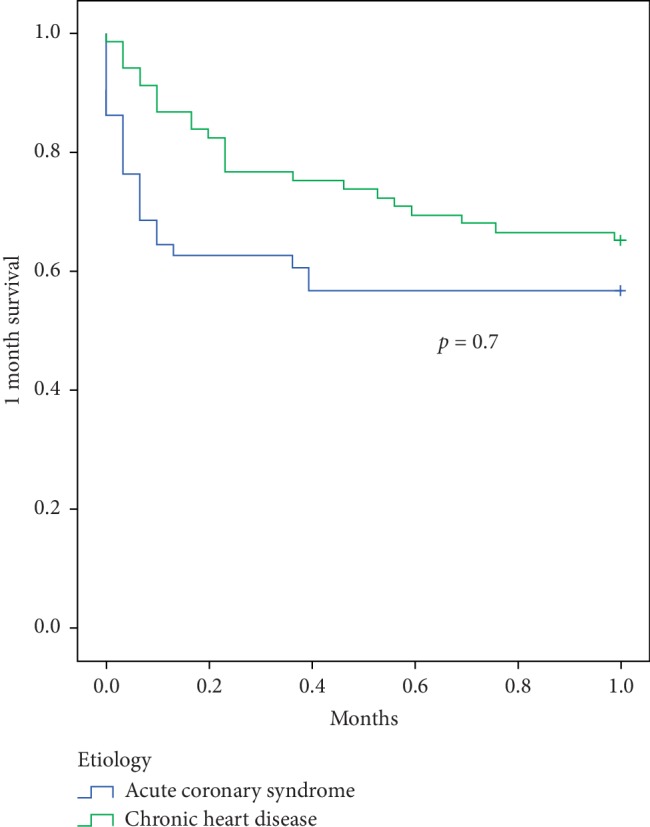
Comparison of Kaplan–Meier survival curves between CS related to ACS and CHF at 1 month. This analysis shows a similar mortality during the first month post-CS between the 2 groups.

**Figure 3:**
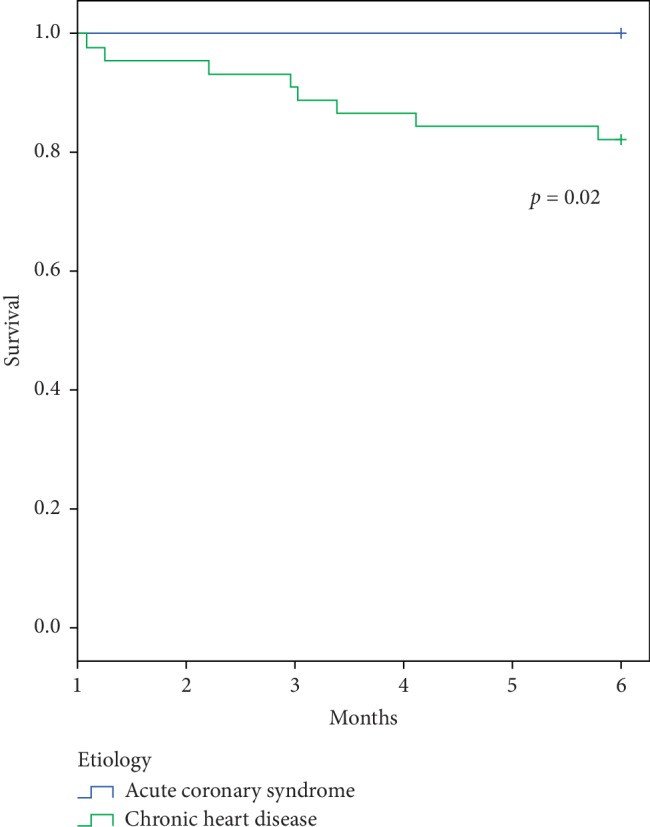
Comparison of Kaplan–Meier survival curves between CS related to ACS and CHF from 1 to 6 months (landmark analysis). This analysis shows a higher mortality between 1 and 6 months in patients with CHF complicated by a first occurrence of CS compared with ACS complicated by CS in survivors after the first month.

Overall, the survival rate was 60.5% at 1 month and 46.7% at 6 months (Table 4). We compared the clinical outcome of the 2 main etiologies of CS in our cohort. We observed that all-cause mortality was similar between patients with ACS and patients with CHF at 1 month (43% vs. 35%; *p*=0.7, respectively) (Figure 2). However, in a landmark analysis of mortality between 1 and 6 months, we observed a significantly higher rate of mortality in patients with CS secondary to an acute decompensation of CHF compared with patients with CS secondary to an ACS (18% vs 0%; *p*=0.01) (Figure 3). In addition, patients surviving a first episode of CS related to acute decompensation of CHF had a higher rate of CS recurrences compared with patients with ACS (13% vs. 0%; *p*=0.01).

## 4. Discussion

In this monocenter registry in a secondary center without surgical back-up, we observed that CS is a common clinical presentation with more than 150 patients admitted in 1 year. This relatively high number of patients admitted to our secondary center is in line with the recent study by Puymirat et al. and underlies the increased prevalence of CS in intensive care units and the critical role of secondary centers in the care of these patients [1]. In addition, the overall mortality in our secondary center was similar to that reported in previous registries and recent randomized trials despite the lack of surgical back-up [1–4, 8]. These results are original and position secondary centers as legitimate and efficient centers for the management of these patients within regional networks centered on tertiary centers, as recently proposed [9].

In this registry, the main etiology of CS was acute decompensation of CHF. Among these patients, chronic ischemic heart disease was the most frequent underlying cardiopathy. Patients with CS secondary to an ACS are not representative of the more frequent clinical presentations. Among patients with CS related to ACS, STEMI was the most frequent origin. Together, CHF and ACS accounted for the vast majority of CS, with the other etiologies representing less than 20% of patients. These results are original and of clinical interest. In the CARDSHOCK registry, the nonischemic cause of CS was rare, accounting for less than 20% of etiologies. In the more recent Japanese registry, ACS accounted for 51% of CS [4]. The difference with our cohort may be related to the definition of CS, a selective skew related to the participating centers or a quick change in the scope of CS, especially a strong improvement in ACS management (early reperfusion) as suggested by the results of the FAST-MI registries [5, 10, 11]. Accordingly, in our contemporary cohort, ACS does not represent the main etiology of CS, but rather the second most common cause. These findings may also be related to the improved treatment of chronic heart failure, which has resulted in a higher number of patients with this condition; consequently, there has been an increase in the number of patients at risk for CS secondary to acute decompensation of CHF [12]. Because acute decompensation of CHF seems to have become the most common cause of CS, it should be a primary target of research.

Although 1-month mortality was similar in CS following ACS or CHF, the long-term survival differed depending on the etiology. In fact, when comparing the prognosis of these 2 main etiologies of CS, we observed that patients with CS secondary to acute decompensation of CHF had a worse prognosis with a higher mortality at 6 months follow-up. This finding is in line with the relatively good prognosis of CS following ACS after the first month and the overall high rate of mortality and recurrences in CHF [8, 13]. Together, our results on the current etiologies and outcome of CS are of clinical interest and better delineate the profile of patients admitted for CS. Our findings confirm the very high mortality of CS during the first month, which is the main target of current research in the field. A recent expert consensus suggested that early and wide use of mechanical circulatory support may potentially improve early survival [9]. Our findings underline the fact that patients surviving the first month after CS related to an acute decompensation of CHF have a poor prognosis with a high mortality and recurrence rate. These patients should therefore be the focus of research efforts. Improvements may be provided by innovative therapeutic options or by monitoring strategies. More aggressive therapies, including up-to-date medical treatment with sacubitril-valsartan, resynchronization, or monitoring with a wireless implantable hemodynamic monitoring (W-IHM) system, may be of interest in patients surviving CS secondary to CHF [12–14]. In addition, a more aggressive use of long-term assistance or transplant may also be of interest in eligible patients [6, 15].

The present study has some limitations. In particular, it is monocentric. However, our institution is a major emergency center and lacks surgical back-up, which prevents a selective skew resulting from the inclusion of patients on the transplant list or those who have been referred for potential mechanical support. In addition, we selected patients with a first occurrence of CS, thus further preventing a selective skew. Finally, the results are in line with the trend in disease outcome for ACS and heart failure.

## 5. Conclusion

In a secondary center without surgical back-up, acute decompensation of chronic heart failure was the most common cause of CS. In addition, we observed that the long-term outcome of these patients is worse than that of patients with CS following an ACS. Overall outcome of patients admitted for CS in secondary centers are similar to that in the literature. Future trials on CS should target patients with ADCHF and aim to improve long-term outcomes through better monitoring and greater access to long-term cardiac assistance.

## Figures and Tables

**Table 1:** Baseline characteristics.

Baseline characteristics (%)	
*Demography and CV risk factors*	
Age (yrs) (mean ± IQR)	71.97 ± 12.75
Male sex	73
HTN	59
Obesity	7.2
Active smoking	22.4
Diabetes	34.2

*Echocardiography*	
LVEF (%) (mean ± IQR)	26.5 ± 10.48

*Comorbidities* (%)	
History of CABG	7.9
History of myocardial infarction	27
History of chronic heart failure	45.4
Peripheric arterial disease	18.4
Chronic kidney disease	19.1
Obstructive chronic bronchitis	10.5
Neoplasia	12.5
ICD	21.7
CRT-P/D	9.2

*Biological characteristics (mean* *±* *IQR)*	
Mean arterial pressure (mmHg)	62 ± 11

**Table 2:** Medical therapy and interventions.

Medical therapy and interventions (%)	
*Drugs*	
Epinephrine	27
Norepinephrine	52.6
Dobutamine	90.8
Diuretics	69.7

*Interventions*	
Coronary angiography	39.5
Urgent revascularisation	27.6
Mechanical circulatory support	5.9
Invasive ventilation	34.1
Dialysis	11.2
Noninvasive ventilation	15.9
Intra-aortic balloon pump	3.3

*Length (days) (mean* *±* *IQR)*	
Duration of inotropics support	7.8 ± 6.8
Duration of intensive care unit stay	9.3 ± 7.5
Duration of resuscitation unit stay	9.7 ± 8.7

**Table 3:** In-hospital complications.

In-hospital (%)	
Sepsis	43.4
Anemia	3.9
Transfusion	8.6
Acute renal failure	32.2
Pericardial effusion	1.3
Stroke	3.3
Major bleeding	4.6

**Table 4:** Clinical outcome.

Clinical outcome (%)	
1-month death	**39.5**
Refractory CS	43.2
Multivisceral dysfunction	28.4
Sepsis	5.2
Cardiac arrest	12.2
Unknown	11
6-month death	**46.7**
6-month recurrence of CS	**7.2**

## Data Availability

The data used to support the findings of this study have not been made available because they are currently being used for additional research purpose.

## Abbreviations

ACS: Acute coronary syndrome
CHF: Chronic heart failure
CS: Cardiogenic shock
LV: Left ventricle.

## References

[1] Puymirat E., Fagon J. F., Aegerter P. (2017). Cardiogenic shock in intensive care units: evolution of prevalence, patient profile, management and outcomes, 1997–2012. *European Journal of Heart Failure*.

[2] Léopold V., Gayat E., Pirracchio R. (2018). Epinephrine and short-term survival in cardiogenic shock: an individual data meta-analysis of 2583 patients. *Intensive Care Medicine*.

[3] Harjola V.-P., Lassus J., Sionis A. (2015). Clinical picture and risk prediction of short-term mortality in cardiogenic shock. *European Journal of Heart Failure*.

[4] Ueki Y., Mohri M., Matoba T. (2016). Characteristics and predictors of mortality in patients with cardiovascular shock in Japan—results from the Japanese circulation society cardiovascular shock registry. *Circulation Journal*.

[5] Puymirat E., Simon T., Cayla G. (2017). Acute myocardial infarction: changes in patient characteristics, management, and 6-month outcomes over a period of 20 years in the Fast-MI program (French registry of acute ST-elevation or non-ST-elevation myocardial infarction) 1995 to 2015. *Circulation*.

[6] Rab T., Ratanapo S., Kern K. B. (2018). Cardiac shock care centers: JACC review topic of the week. *Journal of the American College of Cardiology*.

[7] Thiele H., Schuler G., Neumann F.-J. (2012). Intraaortic balloon counterpulsation in acute myocardial infarction complicated by cardiogenic shock: design and rationale of the intraaortic balloon pump in cardiogenic shock II (IABP-SHOCK II) trial. *American Heart Journal*.

[8] Thiele H., Akin I., Sandri M. (2018). One-year outcomes after PCI strategies in cardiogenic shock. *New England Journal of Medicine*.

[9] Bonello L., Delmas C., Schurtz G. (2018). Mechanical circulatory support in patients with cardiogenic shock in intensive care units: a position paper of the “Unité de Soins Intensifs de Cardiologie” group of the French Society of Cardiology, endorsed by the “Groupe Athérome et Cardiologie Interventionnelle” of the French Society of Cardiology. *Archives of Cardiovascular Diseases*.

[10] Jeger R. V., Radovanovic D., Hunziker P. R. (2008). Ten-year trends in the incidence and treatment of cardiogenic shock. *Annals of Internal Medicine*.

[11] Puymirat E. (2012). Association of changes in clinical characteristics and management with improvement in survival among patients with ST-elevation myocardial infarction. *JAMA*.

[12] McMurray J. J., Packer M., Desai A. S. (2014). Angiotensin-neprilysin inhibition versus enalapril in heart failure. *The New England Journal of Medicine*.

[13] Singh M., White J., Hasdai D. (2007). Long-term outcome and its predictors among patients with ST-segment elevation myocardial infarction complicated by shock. *Journal of the American College of Cardiology*.

[14] Abraham W. T., Adamson P. B., Bourge R. C. (2011). Wireless pulmonary artery haemodynamic monitoring in chronic heart failure: a randomised controlled trial. *The Lancet*.

[15] Jacob K. A., Buijsrogge M. P., Ramjankhan F. Z. (2017). Left ventricular assist devices for advanced heart failure. *New England Journal of Medicine*.

